# Supplementary material for: Candida albicans Reactive Oxygen Species (ROS)-Dependent Lethality and ROS-Independent Hyphal and Biofilm Inhibition by Eugenol and Citral
Source: Microbiol Spectr. 2022 Nov 17;10(6):e03183-22. doi: 10.1128/spectrum.03183-22 (PMC9769929; doi:10.1128/spectrum.03183-22)
Supplement: Supplemental file 1 — Fig. S1 to S6. Download spectrum.03183-22-s0001.pdf, PDF file, 0.8 MB [file spectrum.03183-22-s0001.pdf]

SUPPLEMENTAL MATERIAL

Eugenol							
1000	0.03	0.02	0.01	0	0	0	0
454.5	0.49	0.29	0.21	0.19	0.12	0	0
206.6	0.95	0.70	0.72	0.51	0.44	0.17	0
µg/ml	2.3	5.0	10.9	24.0	52.9	116.4	256
	Citral						

FIG. S1 Checkerboard microtiter plate assay shows the impact of eugenol and citral on *C. albicans* RSY150. The assay revealed a 2 fold reduction of MIC for both EOCs in the presence of the other. Dark grey is oil concentration, light grey is visible growth and white is no growth (OD_{600}).

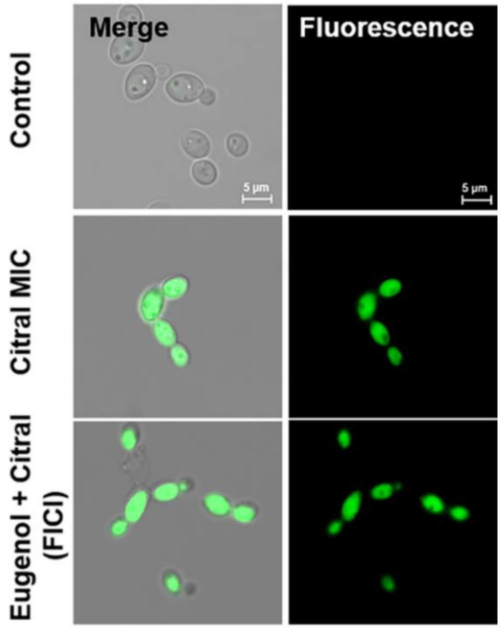

FIG. S2 Citral induces ROS formation in *C. albicans* RBY1132. Representative merged bright field and fluorescence (left) and fluorescence images (right; λ_{ex} = 485 nm; λ_{em} = 528 nm) of *C. albicans* strains exposed to citral at MIC and eugenol-citral at FICI show intracellular ROS accumulation within 24 h. Scale bars are 5 µm for controls, and applicable to all images.

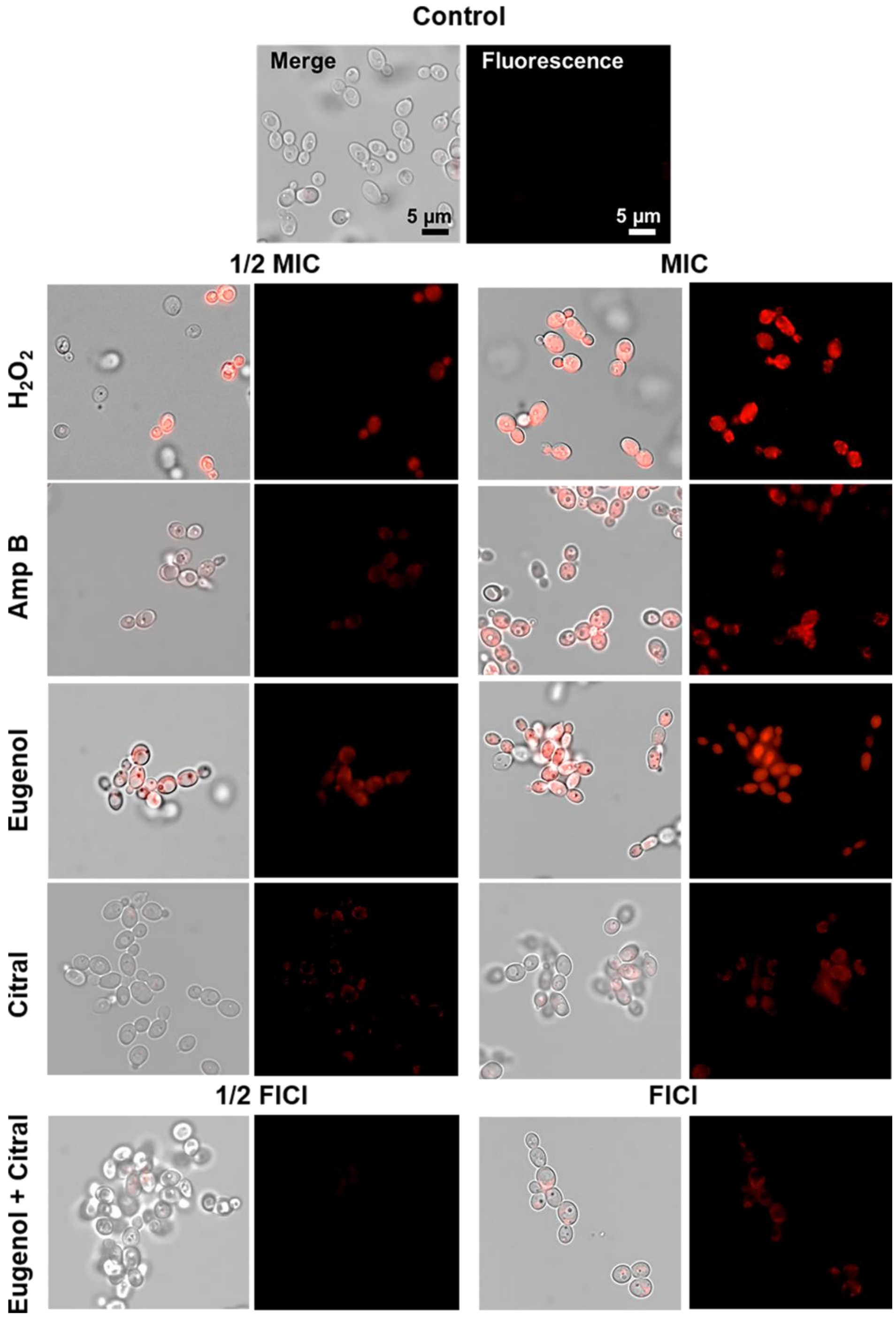

FIG. S3 Impact of eugenol and citral on *C. albicans* RBY1132 cell membrane integrity. Representative merged bright-field/fluorescence (left) and fluorescence (right) images (λ_{ex} = 493 nm; λ_{em} = 636 nm) of treated *Candida* show PI uptake as compared to none for control. Scale bars are 5 µm for controls, and applicable to all images.

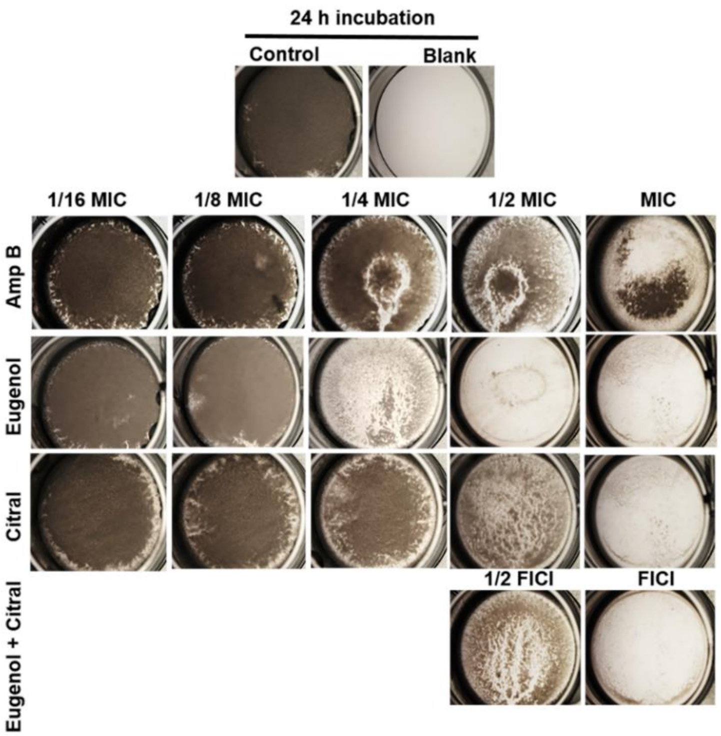

FIG. S4 Eugenol and citral reduce biofilm formation in *C. albicans* RSY150. Representative stereoscopic bright field images show the visual difference between biofilm mass of control cells compared to those treated with eugenol, citral (from 1/16 MIC to MIC) and the two at 1/2 FICI and FICI.

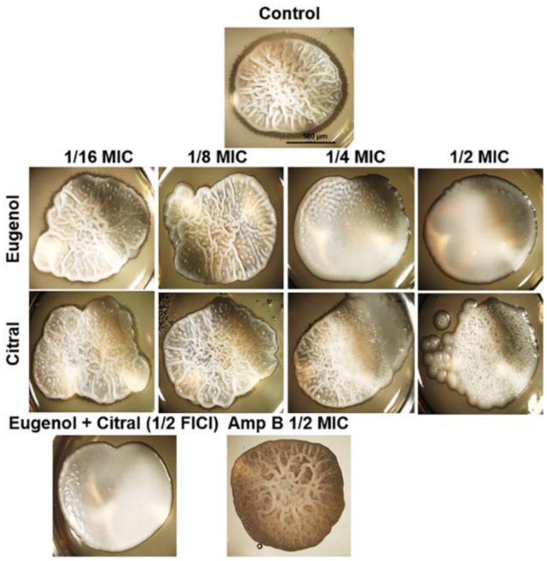

FIG. S5 Eugenol and citral hinder morphological switching in *C. albicans* RSY150. Representative bright field stereoscopic images show *Candida* colonies after 6 d incubation on spider media containing eugenol and citral (from 1/2 MIC to 1/16 MIC) and the two at 1/2 FICI to wrinkled with reduced mycelia. Bar is 500 µm, applicable to all images.

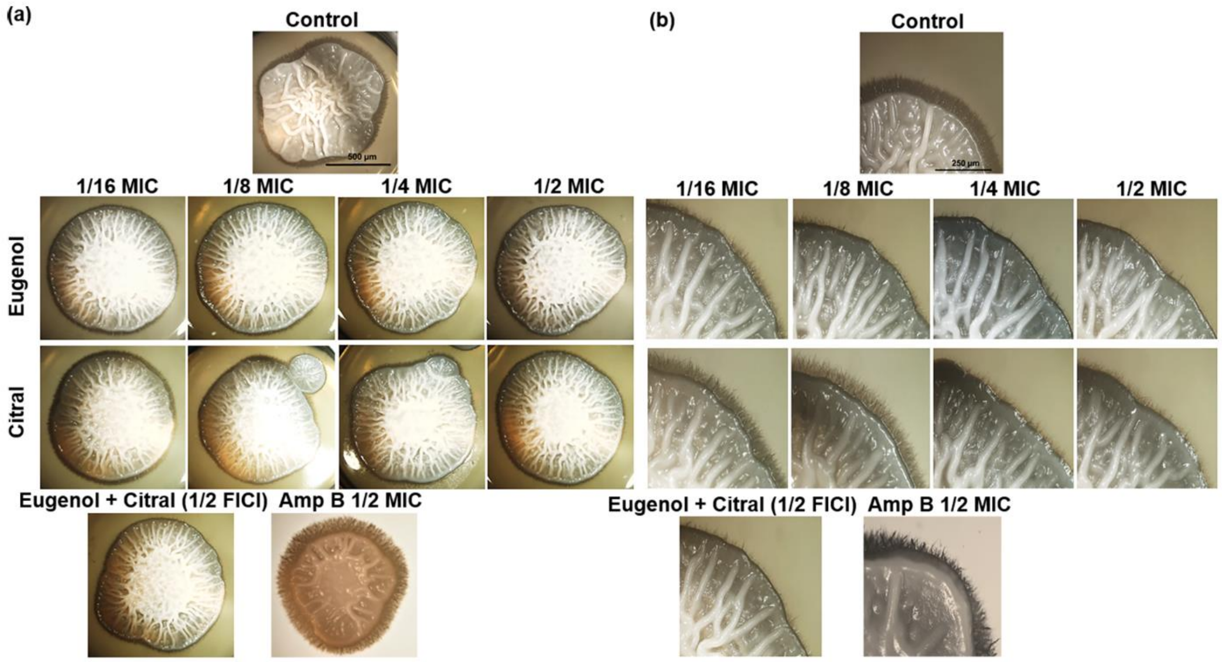

(C)

Relative amount of mycelium					
		1/16 MIC	1/8 MIC	1/4 MIC	1/2 MIC
Control	++++				
Eugenol		+++	++	++	+
Citral		++++	++++	+++	++
1/2 FICI	++				
Amp B					++++

FIG. S6 Impact of pre-treatment with eugenol and citral on *C. albicans* RSY150 and its mycelial morphology. (a,b) Representative bright-field stereoscopic images show the impact of pre-treating *C. albicans* strains with eugenol, citral and the two at their FICI on mycelium formation, for which the symbols in (c), +, ++, +++, ++++, indicate the relative amount of mycelial growth. Bar is 250 (a) and 500 (b) µm and applicable to all images.
